# Supplementary material for: From sequence to enzyme mechanism using multi-label machine learning
Source: BMC Bioinformatics. 2014 May 19;15:150. doi: 10.1186/1471-2105-15-150 (PMC4229970; doi:10.1186/1471-2105-15-150)
Supplement: Additional file 2 — Java code of ml2db. Additional file ml2db_code.tar.gz contains the Java source code to run the multi-label machine learning experiments and save the results to database. The code’s Javadoc is included. [file 1471-2105-15-150-S2.zip › additional file 2/ml2db/utils/doc/serialized-form.html]

Serialized Form


JavaScript is disabled on your browser.


- Overview
- Package
- Class
- Use
- Tree
- Deprecated
- Index
- Help

- Prev
- Next

- Frames
- No Frames

- All Classes

# Serialized Form

- ## Package cern.colt

  - ### Class cern.colt.PersistentObject extends java.lang.Object implements Serializable
- ## Package cern.jet.random

  - ### Class cern.jet.random.AbstractContinousDistribution extends AbstractDistribution implements Serializable
  - ### Class cern.jet.random.AbstractDistribution extends PersistentObject implements Serializable

    - ### Serialized Fields

      - #### randomGenerator

        ```
        RandomElement randomGenerator
        ```
  - ### Class cern.jet.random.Pareto extends AbstractContinousDistribution implements Serializable

    - ### Serialized Fields

      - #### pwr

        ```
        double pwr
        ```
      - #### scale

        ```
        double scale
        ```
      - #### shape

        ```
        double shape
        ```
- ## Package cern.jet.random.engine

  - ### Class cern.jet.random.engine.MersenneTwister extends RandomEngine implements Serializable

    - ### Serialized Fields

      - #### mt

        ```
        int[] mt
        ```
      - #### mti

        ```
        int mti
        ```
  - ### Class cern.jet.random.engine.RandomEngine extends RandomSeedable implements Serializable
- ## Package edu.cornell.lassp.houle.RngPack

  - ### Class edu.cornell.lassp.houle.RngPack.RandomElement extends PersistentObject implements Serializable

    - ### Serialized Fields

      - #### BMoutput

        ```
        double BMoutput
        ```
      - #### BMoutputAvailable

        ```
        boolean BMoutputAvailable
        ```
  - ### Class edu.cornell.lassp.houle.RngPack.RandomJava extends RandomElement implements Serializable
  - ### Class edu.cornell.lassp.houle.RngPack.RandomSeedable extends RandomElement implements Serializable
  - ### Class edu.cornell.lassp.houle.RngPack.RandomShuffle extends RandomElement implements Serializable

    - ### Serialized Fields

      - #### deck

        ```
        double[] deck
        ```
      - #### decksize

        ```
        int decksize
        ```
      - #### generatorA

        ```
        RandomElement generatorA
        ```
      - #### generatorB

        ```
        RandomElement generatorB
        ```
  - ### Class edu.cornell.lassp.houle.RngPack.Ranecu extends RandomSeedable implements Serializable

    - ### Serialized Fields

      - #### iseed1

        ```
        int iseed1
        ```
      - #### iseed2

        ```
        int iseed2
        ```
  - ### Class edu.cornell.lassp.houle.RngPack.Ranlux extends RandomSeedable implements Serializable

    - ### Serialized Fields

      - #### diagOn

        ```
        boolean diagOn
        ```
      - #### in24

        ```
        int in24
        ```
      - #### kount

        ```
        int kount
        ```
      - #### mkount

        ```
        int mkount
        ```
      - #### i24

        ```
        int i24
        ```
      - #### j24

        ```
        int j24
        ```
      - #### iseeds

        ```
        int[] iseeds
        ```
      - #### isdext

        ```
        int[] isdext
        ```
      - #### next

        ```
        int[] next
        ```
      - #### luxlev

        ```
        int luxlev
        ```
      - #### nskip

        ```
        int nskip
        ```
      - #### inseed

        ```
        int inseed
        ```
      - #### jseed

        ```
        int jseed
        ```
      - #### seeds

        ```
        float[] seeds
        ```
      - #### carry

        ```
        float carry
        ```
      - #### twom24

        ```
        float twom24
        ```
      - #### twom12

        ```
        float twom12
        ```
  - ### Class edu.cornell.lassp.houle.RngPack.Ranmar extends RandomSeedable implements Serializable

    - ### Serialized Fields

      - #### c

        ```
        double c
        ```
      - #### cd

        ```
        double cd
        ```
      - #### cm

        ```
        double cm
        ```
      - #### u

        ```
        double[] u
        ```
      - #### uvec

        ```
        double[] uvec
        ```
      - #### i97

        ```
        int i97
        ```
      - #### j97

        ```
        int j97
        ```
- ## Package uk.ac.standrews.utils.main

  - ### Class uk.ac.standrews.utils.main.BooleanArrayPool extends Pool implements Serializable

    serialVersionUID:
    :   2836542638383998534L
- ## Package uk.ac.standrews.utils.main.database

  - ### Class uk.ac.standrews.utils.main.database.DbConn extends java.lang.Object implements Serializable

    serialVersionUID:
    :   5523331251453400716L

    - ### Serialized Fields

      - #### m\_connectionLog

        ```
        java.lang.String m_connectionLog
        ```

        connection log
      - #### m\_connectionPropertiesFilePath

        ```
        java.lang.String m_connectionPropertiesFilePath
        ```

        The path to the properties file
      - #### m\_databaseName

        ```
        java.lang.String m_databaseName
        ```

        the database name
      - #### m\_databaseType

        ```
        java.lang.String m_databaseType
        ```

        the database type, eg. mysql
      - #### m\_debug

        ```
        boolean m_debug
        ```

        the debug state
      - #### m\_driverName

        ```
        java.lang.String m_driverName
        ```

        the database driver class (JDBC driver)
      - #### m\_host

        ```
        java.lang.String m_host
        ```

        the host server (localhost if local)
      - #### m\_password

        ```
        java.lang.String m_password
        ```

        the database password for the user
      - #### m\_port

        ```
        java.lang.String m_port
        ```

        the communication port (default 3306)
      - #### m\_connection

        ```
        java.sql.Connection m_connection
        ```

        the connection to the database
      - #### m\_url

        ```
        java.lang.String m_url
        ```

        the connection url in the form: jdbc:://:/?user=<...>&password=<...>
      - #### m\_user

        ```
        java.lang.String m_user
        ```

        the database user
  - ### Class uk.ac.standrews.utils.main.database.DbManager extends Manager implements Serializable

    serialVersionUID:
    :   -477555623598081647L

    - ### Serialized Fields

      - #### m\_debug

        ```
        boolean m_debug
        ```
      - #### m\_dbWriter

        ```
        DbWriter m_dbWriter
        ```

        the database recorder
      - #### m\_dbConnection

        ```
        DbConn m_dbConnection
        ```

        the database connection properties
      - #### m\_dbCreator

        ```
        DbCreator m_dbCreator
        ```

        the database creator
      - #### m\_dbReader

        ```
        DbReader m_dbReader
        ```

        the database reader
  - ### Class uk.ac.standrews.utils.main.database.TableCreator extends TableManager implements Serializable

    - ### Serialized Fields

      - #### m\_debug

        ```
        boolean m_debug
        ```
  - ### Class uk.ac.standrews.utils.main.database.TableManager extends DbManager implements Serializable

    - ### Serialized Fields

      - #### m\_table

        ```
        Table m_table
        ```
      - #### m\_tableName

        ```
        java.lang.String m_tableName
        ```
  - ### Class uk.ac.standrews.utils.main.database.TableReader extends TableManager implements Serializable
  - ### Class uk.ac.standrews.utils.main.database.TableRow extends TableManager implements Serializable

    - ### Serialized Fields

      - #### m\_column2Values

        ```
        java.util.TreeMap<K,V> m_column2Values
        ```

        the row values (the keys should be names of table columns)
      - #### m\_isCorrect

        ```
        boolean m_isCorrect
        ```

        whether this row is correct for the table and could be written
      - #### m\_debug

        ```
        boolean m_debug
        ```
  - ### Class uk.ac.standrews.utils.main.database.TableWriter extends TableManager implements Serializable

    - ### Serialized Fields

      - #### m\_debug

        ```
        boolean m_debug
        ```
- ## Package uk.ac.standrews.utils.main.guiutils

  - ### Class uk.ac.standrews.utils.main.guiutils.GuiUtils extends javax.swing.JFrame implements Serializable
- ## Package uk.ac.standrews.utils.main.maputils

  - ### Class uk.ac.standrews.utils.main.maputils.MapOfMaps extends java.util.TreeMap<java.lang.String,java.util.Map<java.lang.String,java.lang.String>> implements Serializable

    serialVersionUID:
    :   1L

    - ### Serialized Fields

      - #### m\_debug

        ```
        boolean m_debug
        ```
      - #### m\_extStringPool

        ```
        StringPool m_extStringPool
        ```
      - #### m\_intStringPool

        ```
        StringPool m_intStringPool
        ```
      - #### m\_valuesStringPool

        ```
        StringPool m_valuesStringPool
        ```
      - #### m\_acceptEmptyStrings

        ```
        boolean m_acceptEmptyStrings
        ```
      - #### m\_internalKeys

        ```
        java.util.TreeSet<E> m_internalKeys
        ```
  - ### Class uk.ac.standrews.utils.main.maputils.OneToMany extends java.util.TreeMap<T,U> implements Serializable

    serialVersionUID:
    :   1L
  - ### Class uk.ac.standrews.utils.main.maputils.OneToManyMap extends OneToMany<T,java.util.Set<U>> implements Serializable

    serialVersionUID:
    :   1L

    - ### Serialized Fields

      - #### t

        ```
        java.lang.Object t
        ```

        generics type for keys
      - #### u

        ```
        java.lang.Object u
        ```

        generics type for values in vector of values
      - #### m\_debug

        ```
        boolean m_debug
        ```

        the map containing for each key the collection of values that key can
        have
- ## Package uk.ac.standrews.utils.main.stats

  - ### Class uk.ac.standrews.utils.main.stats.PseudoTruncatedPareto extends TruncatedPareto implements Serializable

    - ### Serialized Fields

      - #### m\_pseudoRandomNumbers

        ```
        java.lang.Integer[] m_pseudoRandomNumbers
        ```
  - ### Class uk.ac.standrews.utils.main.stats.TruncatedPareto extends Pareto implements Serializable

    - ### Serialized Fields

      - #### m\_offset

        ```
        int m_offset
        ```

        Offset to match other numbering systems. For example, the Java arrays/map
        indexing system starts at 0, while the Pareto extracted integers start at
        2. This conversion will need a -2 offset.
      - #### m\_truncationThreshold

        ```
        int m_truncationThreshold
        ```

        the truncation threshold, no numbers extracted can be above it
- ## Package uk.ac.standrews.utils.main.stringutils

  - ### Class uk.ac.standrews.utils.main.stringutils.Pool extends java.lang.Object implements Serializable

    serialVersionUID:
    :   1297632992006788554L

    - ### Serialized Fields

      - #### m\_pool

        ```
        java.util.concurrent.ConcurrentHashMap<K,V> m_pool
        ```
      - #### m\_size

        ```
        int m_size
        ```
      - #### m\_debug

        ```
        boolean m_debug
        ```
  - ### Class uk.ac.standrews.utils.main.stringutils.StringPool extends Pool implements Serializable

    serialVersionUID:
    :   -6392176353438781631L

- Overview
- Package
- Class
- Use
- Tree
- Deprecated
- Index
- Help

- Prev
- Next

- Frames
- No Frames

- All Classes
